# TSCOT ^+^ Thymic Epithelial Cell-Mediated Sensitive CD4 Tolerance by Direct Presentation 

**DOI:** 10.1371/journal.pbio.0060191

**Published:** 2008-08-05

**Authors:** Sejin Ahn, Gwanghee Lee, Soo Jung Yang, Deokjae Lee, Seunghyuk Lee, Hyo Sun Shin, Min Cheol Kim, Kee Nyung Lee, Douglas C Palmer, Marc R Theoret, Eric J Jenkinson, Graham Anderson, Nicholas P Restifo, Moon Gyo Kim

**Affiliations:** 1 Laboratory of Cellular and Molecular Immunology, National Institute of Allergy and Infectious Diseases, Bethesda, Maryland, United States of America; 2 Research Center for Molecular and Cellular Biology, Inha University, Incheon, Korea; 3 Department of Biological Sciences, Inha University, Incheon, Korea; 4 National Institute of Cancer, National Institutes of Health, Bethesda, Maryland, United States of America; 5 Medical Research Council (MRC) Centre for Immune Regulation, Institute for Biomedical Research, University of Birmingham, United Kingdom

## Abstract

Although much effort has been directed at dissecting the mechanisms of central tolerance, the role of thymic stromal cells remains elusive. In order to further characterize this event, we developed a mouse model restricting LacZ to thymic stromal cotransporter (TSCOT)-expressing thymic stromal cells (TDLacZ). The thymus of this mouse contains approximately 4,300 TSCOT^+^ cells, each expressing several thousand molecules of the LacZ antigen. TSCOT^+^ cells express the cortical marker CDR1, CD40, CD80, CD54, and major histocompatibility complex class II (MHCII). When examining endogenous responses directed against LacZ, we observed significant tolerance. This was evidenced in a diverse T cell repertoire as measured by both a CD4 T cell proliferation assay and an antigen-specific antibody isotype analysis. This tolerance process was at least partially independent of *Autoimmune Regulatory Element* gene expression. When TDLacZ mice were crossed to a novel CD4 T cell receptor (TCR) transgenic reactive against LacZ (BgII), there was a complete deletion of double-positive thymocytes. Fetal thymic reaggregate culture of CD45- and UEA-depleted thymic stromal cells from TDLacZ and sorted TCR-bearing thymocytes excluded the possibility of cross presentation by thymic dendritic cells and medullary epithelial cells for the deletion. Overall, these results demonstrate that the introduction of a neoantigen into TSCOT-expressing cells can efficiently establish complete tolerance and suggest a possible application for the deletion of antigen-specific T cells by antigen introduction into TSCOT^+^ cells.

## Author Summary

T cells play critical roles in the immune response. While developing in the thymus (from whence T cells and their precursors, thymocytes, derive their name), thymocytes are selected for the ability to recognize harmful antigen (positive selection), while those that respond to antigens present in their own body are eliminated (negative selection). Dogma holds that the thymus is divided into different functional compartments to ensure that these contrasting selection processes occur efficiently: the cortex is thought to be responsible for positive selection and the medulla for negative selection. In this study, we made use of a novel transgenic mouse (carrying a LacZ marker in a small fraction of cells in the cortex) to test whether the cortex is really excluded from negative selection. We were able to show that the introduced LacZ “antigen” present only in the cortical cells leads them to eliminate any LacZ-reactive T cells from the immune repertoire and leads to tolerance of the LacZ “antigen” by the body's immune system. This process is highly efficient, such that a relatively tiny number of antigen molecules present in a small fraction of the cells in the thymic cortex can singularly perform proofreading of all developing thymocytes.

## Abstract 3

A new study shows that antigen-specific negative selection of developing thymocytes is a property of thymic cortical epithelial cells, challenging the view that this process requires contact with specialized antigen-presenting cells found in the thymic medulla.

## Introduction

T cell tolerance is established mainly in the thymus where the T cell population develops and learns by a process called negative selection to avoid harmful reactivity against self-antigens expressed in that thymus (reviewed in [1,2]). In the periphery, organ-specific tolerance can be established by various other mechanisms, including anergy [3], ignorance [4], and regulatory T cells [5]. Furthermore, antigen-presenting cells (APC) lacking costimulatory molecules in peripheral tissues initiate abortive immune responses [6].

The thymic microenvironment is organized and equipped to achieve efficient self-tolerance by providing stimulatory signals to developing self-reactive thymocytes. For a diverse T cell repertoire, this negative selection process occurs primarily in the thymic medullary compartment (reviewed in [7,8]). The major player among the hematopoietic cells is the dendritic cell (DC), which possesses a highly efficient antigen presentation capability. In addition, it is widely accepted that thymic medullary epithelial cells (mTEC) that express low levels of tissue-specific peripheral antigens in a promiscuous/ectopic fashion [9,10] can also initiate clonal deletion. Discovery of the *AIRE* gene and its expression in mTEC has led to an understanding of its critical regulatory role in the removal of autoreactive T cells, particularly against tissue-specific antigens expressed in the endocrine system (reviewed in [11,12]). However, AIRE is also expressed in non-mTEC, including thymic DC [13,14] and in cortical thymic epithelial cells (cTEC) from Rag-2–deficient thymus [15]. Furthermore, the cross-presentation pathway can participate in the CD4 and CD8 tolerance for the membrane-bound antigens [16]. Therefore, the natures of cell types responsible for the tolerance induction still remain unsettled.

The role of cortical epithelium in tolerance induction has been controversial (reviewed in [17–20]). Several experiments using thymus transplantation have clearly indicated that thymic epithelium exhibits toleragenic function [21–24]. In contrast, experiments using transgenic mice with targeting of major histocompatibility complex class II (MHCII) [25] or MHCI [26] molecules to the thymic cortical compartment (and the skin) using a fortuitous keratin 14 promoter led to the conclusion that cTEC are not capable of inducing tolerance. Such results have given rise to the idea that the thymic microenvironment is compartmentalized, with positive selection taking place in the cortex and negative selection in the medulla. If this is a true dogma, there will be autoimmune responses to the antigens specifically expressed in the cortical epithelial cells. However, when other antigens were targeted into cTEC using the same promoter, incomplete but significant tolerance to the specific antigens was observed [27,28]. In the case of a circulating antigen (C5), all types of thymic APC, including the cTEC, could effect efficient negative selection in vitro [29]. Finally, the question of the role of circulating peripheral DC in the induction of thymic tolerance has also been raised [30] and tested true [31].

Experiments regarding the ability of cTEC to efficiently present antigens have also been controversial. In early studies, the death of cortical thymocytes upon activation by antibody or peptides was interpreted as resulting from antigen presentation by the cortical stromal cells [32,33]. In addition, a study with purified thymic APC suggested that cTEC were able to present antigens to a self-reactive hybridoma, with an efficiency comparable to that of thymic DC [34]. However, later studies indicated that a cell line with cTEC properties was inefficient in processing antigens both in vitro and in vivo [35,36]. In contrast, Volkmann and his colleagues, using enriched stromal cell preparations from adult thymus, demonstrated that cTEC are able to present soluble antigens as efficiently as DC or mTEC in reaggregate cultures. In many, if not all, of the above studies, however, difficulties in interpretation still persist, in particular, because of a lack of sufficient understanding about the nature of the defined cTEC subpopulation under study, as well as the purity of the cells expressing the specific antigens that were used in the assays. More recently, Gray and colleagues reported that well-defined, purified cTEC, as well as mTEC, express costimulatory molecules and can stimulate naive T cells as much as thymic dendritic cells do in vitro [37]. Therefore, we felt it was necessary to re-evaluate the role of the cTEC subpopulation in central tolerance induction using a different model system, one perhaps better suited to more directly answering the question of whether subpopulation of cTEC can present endogenous antigens and whether this can lead to deletion of thymocytes.

Previously, in an effort to separate thymic epithelial cell (TEC) components, we introduced a new marker (Ly110), designated thymic stromal cotransporter (TSCOT), which is expressed in a specific TEC subpopulation. TSCOT is a putative 12-transmembrane protein, located mainly in the thymic cortex [38]. TSCOT is not expressed in any other tissues, as detected by quantitative reverse-transcription PCR (RT-PCR) [39]. It is also not expressed in thymocytes [38]. TSCOT^+^ thymic stromal cells are all MHCII^+^ and CDR1^+^/6C3^+^, well-defined cortical epithelial markers [40], with observable variations in levels during different developmental stages [41]. In this study, we introduce a new mouse model system called TSCOT delta LacZ (TDLacZ) that expresses a β-galactosidase (β-gal) in the TEC subpopulation. This model system constitutes a new tool for the study of TEC development and function. First, we were able to follow TSCOT-expressing TEC by β-gal activity assays or antibody staining and flow cytometry using an anti-TSCOT monoclonal antibody (mAb) [41]. LacZ enzymatic activity could also be assayed for the location of cells with a high degree of sensitivity, in both sections and the whole organism, and expression could be assessed in a quantitative manner. Second, because the protein is generated by an endogenous promoter, this system is designed to express normal doses of neoself-antigen relative to other competing cellular proteins. This is in contrast to some previous systems for the targeting of cortical expression, in which MHC molecules were displayed at unusually low levels [19,26]. Third, the absence of the TSCOT promoter activity in peripheral tissues precludes the involvement of recirculating DCs, which might deliver peripheral antigens to the thymus, and present them ectopically.

By targeting LacZ protein as a neoantigen within the TSCOT-expressing thymic epithelium, we were able to demonstrate that TSCOT^+^CDR1^+^ TEC alone, without any help from the mTEC or DC, is able to establish deletional tolerance in an AIRE-independent manner with a surprisingly high degree of efficiency.

## Results

### A TDLacZ Mouse Model for TEC Subpopulation-Specific Antigen Expression

**Figure 1:**
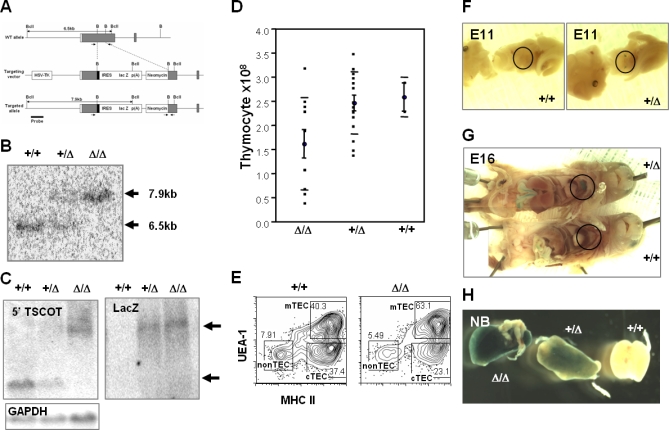
Targeting LacZ into the TSCOT Locus for Expression in Thymic Epithelium (A) Schematic presentation of +/+ (top), the targeting construct (middle), and the targeted allele (bottom). The restriction sites BclI (BclI) and BamHI (B) and location of coding regions are shown. The probe (627 bp) used in the Southern blot is shown as a thick line under the targeted allele. PCR typing primer positions are shown as small arrows. (B) Southern blot of a BclI digest. The +/+ allele is 6.5 kb, and the targeted allele is 7.9 kb. (C) Northern blot for the TSCOT-LacZ fusion message. The probe (TSCOT, LacZ, and GAPDH control) is at the top left. (D) Total thymocyte yields from Δ/Δ, Δ/+, and +/+. Averages and standard deviations are shown. (E) Thymic stromal cell analyses of wild type and homozygotes. CD45^−^ gates of 2-wk-old thymuses are shown for MHCII and UEA-1. The cTEC, mTEC, and nonTEC gates are indicated. (F and G) β-gal activity in the developing thymus (E11 and E16, respectively) of fetuses from Δ/+ and +/+ littermates. Only the targeted thymus was stained (within the black circles). Endogenous β-gal activity was detected in the intestines of both mice. (H) Newborn thymuses from Δ/Δ, Δ/+, and +/+ littermates show a gene dose-dependent expression.

We established a new system by knocking-in the *LacZ* gene into the TSCOT locus between two BamHI sites (Figure 1A). LacZ was transcribed in the same message with the 5′ portion of the TSCOT message, and translation of LacZ was facilitated by incorporating an internal ribosome entry site (IRES) sequence [42]. The targeting was confirmed by Southern blotting (Figure 1B). Northern blotting confirmed that the LacZ message was in a fusion transcript with the 5′ portion of the TSCOT message (Figure 1C).

The TDLacZ mice evidenced no distinguishable abnormalities with regard to thymic structure as the result of the deletion in TM5-TM12 portion of the TSCOT protein. In Figure 1E, we show that the similar thymic stromal patterns of the 2-wk-old homozygote and the wild type. The small difference in the fraction of stromal cell populations was within the experimental variations. There was also no difference detected in the profiles between hetero- and homozygote littermates of the various ages (unpublished data). The N-terminal portion including transmembrane spans 1–4 of the protein still remained expressed on the cell surface, as detected by flow cytometry (unpublished data). The only apparent difference was for the total thymocyte yield at 6 wk of age, which was slightly lower in about one-third of the TDLacZ homozygotes (Figure 1D). However, we failed to detect any reproducible differences in the profiles of thymocyte population except the individual variation. In addition, an analysis of 6-mo-old mice also showed no significant differences detected in the recovery of thymocytes and major profiles of CD25, CD44, CD4, and CD8 (unpublished data). When 5CC7 T cell receptor (TCR) Tg mouse was bred with TDL, no significant differences for the thymocyte populations were found in selecting or nonselecting background (F. Flomerfelt, unpublished data).

When β-gal activity was assessed in TDLacZ mice at embryonic day 11 (E11), the time at which thymus organogenesis is initiated, LacZ was already expressed in the two separated thymic rudiments, but it was not expressed in the wild-type littermates (Figure 1F). This expression was not detected in any other organs. At E16, when the thymus harbors mostly developing double-negative (DN) and double-positive (DP) cells, thymic expression of LacZ also was very clear (Figure 1G). In addition, endogenous β-gal activity appeared in the TDLacZ intestine at E16, as in the wild-type control (unpublished data). β-gal activity in thymus samples from newborn TDLacZ pups showed a gene dose dependency (Figure 1H).

**Figure 2:**
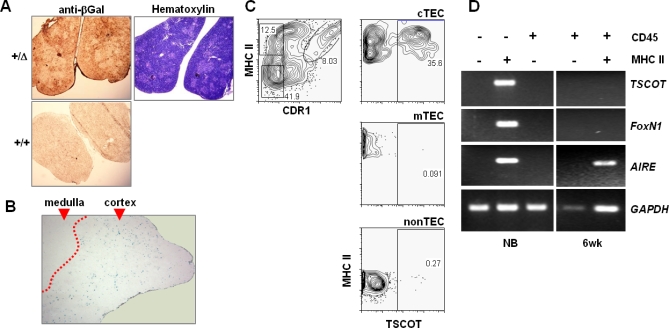
LacZ and TSCOT-Expressing Cells in the Thymus (A) Analyses of the thymus using an antibody against β-gal in newborns from a TDLacZ mouse heterozygote (Δ/+) and a wild type (+/+). Hematoxylin staining is shown on the right. (B) β-gal activity of the thymus from 8-wk-old homozygous knock-in mouse (Δ/Δ) at 10× magnification. Cortical and medullary areas are indicated, and the boundary between LacZ stained and unstrained areas is artificially marked as a dotted line for better visualization. (C) Flow cytometric analysis of TSCOT-expressing cells using the markers in total thymic CD45^−^ stromal cells. The profiles of cortical marker CDR1 and medullary marker UEA-1. Defined cTEC, mTEC, and nonTEC are gated. Each gated population is shown as TSCOT and UEA-1 levels on the right. Fraction of TSCOT^+^ cells are shown in percentages. (D) Message expression of TSCOT, FoxN1, AIRE, and GAPDH by sorted thymic compartments according to CD45/MHCII status using RT-PCR (30 cycles). The cells were isolated either from newborns or 6-wk-old thymuses, and the cell surface markers used for sorting are shown on top.

We next located the LacZ-expressing cells in thymic sections. At the newborn stage, anti-LacZ antibody staining revealed the expression mostly in the thymic cortex as expected (Figure 2A). When the thymus had fully matured (8 wk of age), LacZ activity was also detected in the cortex (Figure 2B). This is consistent with our previous result that TSCOT protein and mRNA expression was located in the cortex [38]. After careful examination, we occasionally found LacZ staining extends to corticomedullary junction (unpublished data and see later). In an attempt to characterize the TSCOT-expressing cells in the mature thymus in greater detail, flow cytometric analysis was conducted using a TSCOT-specific mAb. Previously, we group the thymic stromal cell populations into at least five different subpopulations [43]. Three main population are cTEC as CDR1^+^UEA-1^−^MHCII^hi^G8.8^+^, mTEC as CDR1^−^UEA-1^+^MHCII^hi^ or MHCII^med^G8.8^+^, as well as nonepithelial population, nonTEC, CDR1^−^UEA-1^−^MHCII^−^G8.8^−^. As shown in Figure 2C, 35.6% of cTEC (CDR1^+^MHCII^hi^) population expresses TSCOT, whereas none of the mTEC or nonTEC population expresses detectible levels of TSCOT. Although all of the TSCOT-expressing cells were positive for cortical marker CDR1 [41], a fraction of TSCOT^+^CDR1^+^ cells were found to express UEA-1 (unpublished data and see Discussion). Finally, we examined whether TSCOT mRNA was expressed along with FoxN1 and AIRE mRNAs (Figure 2D). TSCOT and FoxN1 were detectable only in the MHCII^+^CD45^−^ epithelial compartment. In contrast, the AIRE message was detectable in both the epithelial and CD45^+^MHCII^+^ compartments as expected, supporting the previous result on the expression in hematopoietic stromal cells of the thymus [13–15].

### Quantitative Aspects of LacZ Expression in TDLacZ Mouse

**Figure 3:**
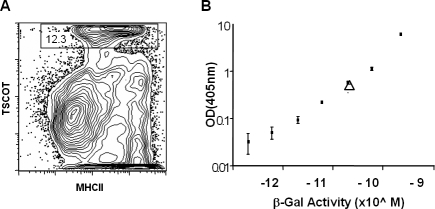
Estimation of the Number of LacZ Molecules Expressed in the TSCOT-Expressing Cells (A) Flow cytometric analysis of the percentage of TSCOT-expressing cells in the thymus preparation. (B) Standard curve of β-gal enzyme activity using purified LacZ protein. The purified LacZ enzyme from the commercial source was measured by the weight, and the stock enzyme solution was diluted serially to generate the curve. The open triangle indicates the measured value for a protein extract generated from 30 TDLacZ homozygous (Δ/Δ) thymuses.

Next, in order to measure sensitivity of tolerance induction, we estimated the average quantity of antigen expressed in one adult thymus by measuring the β-gal activity of the LacZ protein in purified thymic stromal cells. We isolated the cells from TDLacZ^Δ/Δ^ mice, and stained them with a mAb against TSCOT (Figure 3A). In this preparation using 28 animals, TSCOT^+^ cells (12.3%) corresponded to 1.2 × 10^5^ cells. This calculates out to a total of about 4,300 TSCOT^+^ cells per thymus. When this cell preparation was lysed and the β-gal activity was evaluated (Figure 3B, and unpublished data), we were able to determine the LacZ concentration from a standard curve (2 × 10^−11^ M of 50-μl reactions). These numbers corresponded to 5,017 molecules of LacZ protein per TSCOT^+^ cell by the simple mathematical calculation of concentration × volume × Avogadro number/cell number; 2× 10^−11^ M × 50/10^6^ × 6.02 × 10^23^ molecules in 1.2 × 10^5^ cells in the experiment shown. In the second experiment, the final number was 6,825 molecules per cell.

### Can Antigen Expressed in TSCOT^+^ TEC Elicit Self-Tolerance?

**Figure 4:**
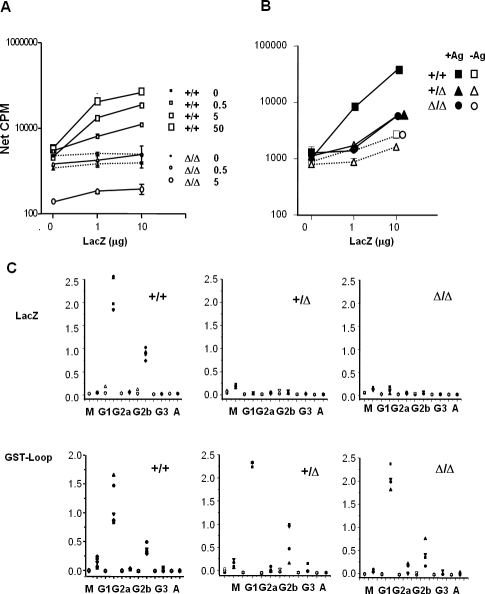
CD4^+^ Tolerance to LacZ in the TDLacZ Mouse (A) CD4^+^ T cells of mice immunized with 0, 0.5, 5, or 50 μg of LacZ, were stimulated with 0, 1, or 10 μg/ml of LacZ. The responses of LacZ immunized mice in solid lines with open symbols, the adjuvant immunized mice in dotted lines with closed symbols. Rectangles indicate +/+, and ovals Δ/Δ. (B) The T cells response of +/+ (rectangles), Δ/+ (triangles), and Δ/Δ (ovals) immunization with (solid lines and closed symbols) or without (dotted lines and open symbols) 25 μg of LacZ. There were no significant differences in the recovery of cell numbers from the immunized mice of any genotypes. (C) Antibody isotype profiles of +/+, +/Δ, and Δ/Δ mice immunized with LacZ (upper panels) or the GST-TSCOT-Loop (lower panels). The OD reading of individual serum is shown as a single symbol. Open symbols are for preimmune serum, and filled symbols are for immunized serum. IgM (M), IgG1 (G1) IgG2a (G2a), IgG2b (G2b), IgG3 (G3), and IgA (A) levels are shown.

TDLacZ and wild-type animals were immunized with recombinant LacZ protein and examined for a LacZ-specific polyclonal CD4^+^ T cell response. As shown in Figure 4A, a concentration-dependent LacZ-induced proliferative response was detected in purified CD4^+^ lymph node T cells from wild-type B6 animals, whereas T cells from TDLacZ mice clearly showed no response to LacZ. Both the heterozygous and homozygous animals showed a large reduction in proliferation (Figure 4B). LacZ-specific antibody responses were then evaluated by ELISA (Figure 4C). When whole LacZ protein was administered in CFA, wild-type mice produced both IgG1 and IgG2b isotypes specific for LacZ. In contrast, heterozygous and homozygous TDLacZ mice did not produce such antibodies (Figure 4C, top). In order to assess the possibility that this represented tolerance at the B cell level, we administered a GST-tagged loop portion of TSCOT (GST-Loop) in CFA and screened for specific antibody responses with a His-tagged loop protein (His-Loop) in an ELISA. In this case, with help provided by T cells specific for GST, both the heterozygous and homozygous TDLacZ mice made as much anti-loop IgG1 and IgG2b antibodies as the wild-type mice (Figure 4C, bottom). These results clearly show that the presence of LacZ expression in the subpopulation of TSCOT^+^ TEC was sufficient for the tolerization of LacZ-specific CD4^+^ T cells, and this tolerance is not due to the absence of whole TSCOT molecules in the animal.

### Molecules Involved in TSCOT^+^ TEC-Mediated Tolerance

**Figure 5:**
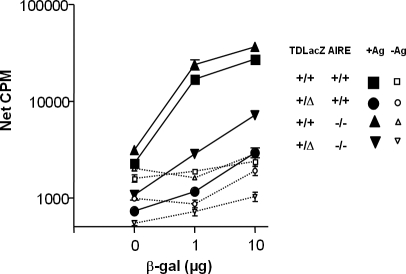
AIRE Is Not Necessary for Tolerance to Antigens Expressed by TSCOT^+^ TECs Proliferation assays in response to β-gal for lymph node CD4^+^ T cells from LacZ-immunized (10 μg) wild-type (rectangles), TDLacZ (ovals), or AIRE-deficient (triangles), or from AIRE-deficient × TDLacZ (inverted triangles) mice. The responses of the LacZ-immunized mice (+Ag) are shown as solid lines with closed symbols, while those of the adjuvant-only immunized mice (−Ag) are shown as dotted lines with open symbols.

Because AIRE is known to play a key role in the establishment of tolerance to antigens promiscuously/ectopically expressed in small amounts by mTEC [44–46], we investigated the possible role of AIRE in TSCOT^+^ TEC with regard to the induction of tolerance. We crossed the TDLacZ mouse with an AIRE-deficient mouse, and conducted the same proliferation assay for an anti-LacZ CD4^+^ T cell response to the LacZ protein. As shown in Figure 5, the AIRE-deficient mice displayed slightly enhanced anti-LacZ responses compared to the wild type, possibly due to the introduced cross-reactivity or autoreactivity. When one copy of LacZ was expressed by breeding the AIRE knock-out (KO) to the TDLacZ mouse, the anti-LacZ-specific proliferative response was clearly reduced. In the specific responses to 1 μg of antigen, the degrees of responses contributed by AIRE were similar (difference between wild type and AIRE KO vs. that between TDLacZ to TDLacZ AIRE KO). These results strongly suggest the presence of another pathway that AIRE does not play a major role in the induction of tolerance to an antigen expressed in TSCOT^+^ TEC.

**Figure 6:**
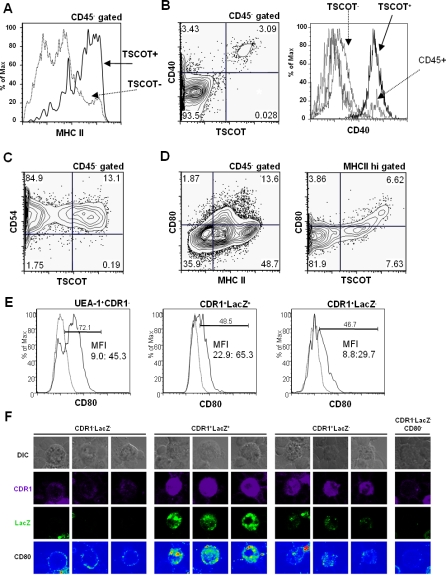
Costimulatory Molecules and Other Surface Marker Profiling of TSCOT-Expressing Cells (A) MHCII profiles of TSCOT^+^ cells (solid line) and TSCOT^−^ cells (dotted line). (B) CD40 profile of CD45^−^ gate on the left, CD40. Histograms of TSCOT^+^, TSCOT^−^(mTEC+cTEC), and CD45^+^ are indicated on the right. Background histogram of live gate is shown but not indicated. (C) CD54 profile of CD45^−^ gate. (D) CD80 profile of CD45^−^ gate and MHCII ^hi^ gate. (E) Histogram of CD80 levels in mTEC (UEA-1^+^CDR1^−^), TSCOT^+^cTEC(CDR1^+^LacZ^+^), TSCOT^−^cTEC (CDR1^+^LacZ^−^). Specific CD80 stain (solid line) and background (dotted line) of the same gates are shown. Percentage of positive cells in each gates, MFI of negative and positive gates are indicated. (F) CD80 expression pattern of selected CDR1 and LacZ stained TDLacZ stromal cells. DIC, CDR1, LacZ, and CD80 staining patterns are shown.

We further assessed the presence or absence of selected costimulatory and adhesion molecules in the TSCOT-expressing cells. Although there has been reports that cortical epithelium does not express costimulatory molecule by histological analysis, we had reasons to believe that this conclusion may be false based on our observation of disparity between histology and flow cytometry [47]. As shown in Figure 6A–6C, flow cytometry revealed that TSCOT^+^ cells are all positive for MHCII, CD40, and CD54 expression. In more detailed analysis, the relative CD40 level of TSCOT^+^ cells was similar to that of some CD45^+^ cells (presumably dendritic cells), and higher than TSCOT^−^ cells that contain TSCOT^−^cTEC and mTEC populations (Figure 6B, histogram). An important costimulatory molecule, CD80 was expressed in some TSCOT^+^ cells as shown in Figure 6D (CD45^−^ gate and CD45^−^MHCII^hi^ gate where most of the TSCOT^+^ cells reside). In order to compare the relative levels of CD80 between mTEC and TCSOT^+^ cells, the multiparameter analyses in flow cytometry and in confocal microscopy were applied including LacZ staining with the stromal cells prepared from TDLacZ thymus. As seen in (Figures 6E and 6F, and S1). In both analyses, mean fluorescence intensity (MFI) of the relative levels of CD80 was higher in TSCOT^+^cTEC (CDR1^+^LacZ^+^) than in other cells mTEC (UEA-1^+^CDR1^−^) and TSCOT^−^cTEC (CDR1^+^LacZ^−^). CD86 was not detected under our conditions, possibly due to the trypsin-sensitive nature of this marker (unpublished data). These results clearly suggest that TSCOT^+^ cells can function as efficient APC.

### Complete Deletion of Monoclonal TCR Transgenic Thymocytes Specific for LacZ at the DN Stage in the Presence of TDLacZ Epithelium

**Figure 7:**
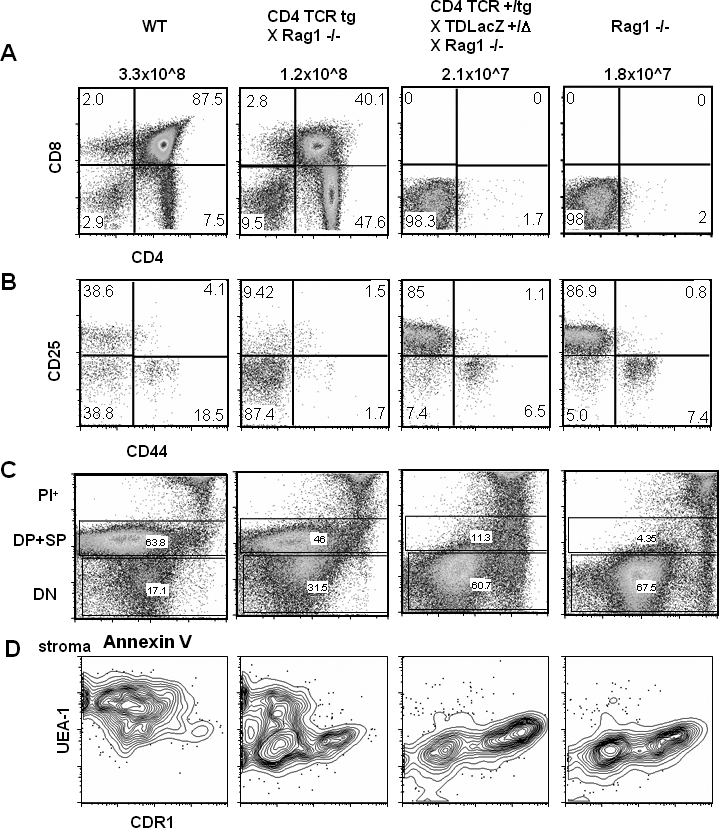
Anti-LacZ–Specific TCR-Bearing Transgenic Thymocytes Are Deleted at the DN Stage in the Presence of TSCOT-LacZ Expression (A) CD4 and CD8 thymocyte profiles. The names of each of the four mouse strains used and their total thymocyte yields are shown above the plots. The percentages of the single and double staining subsets are shown in the four quadrants. (B) CD25 and CD44 profiles of the DN thymocytes with percentages in the quadrants. (C) Annexin V staining of all cells stained with CD4, CD8, and PI shown in FL3 channel. PI^+^ cells are at the top, DP and SP cells in the middle, and DN cells in the bottom. (D) The UEA-1 and CDR1 profiles of the CD45^−^ stromal cell compartment from the four different mouse lines. The genotypes of each mice are verified for the loci of Rag1, TDL, and TCR α and β chains by PCR

To determine the specific mechanism for the observed tolerance, we utilized a monospecific TCR Tg mouse, BgII (D. Palmer, Marc R. Theoret, and N. Restifo, unpublished data; see Materials and Methods) that carries an anti-LacZ TCR transgene from a CD4^+^ LacZ-specific T cell clone [48]. This line was crossed with Rag1^−/−^ to establish a monospecific TCR-bearing T cell population. When the BgII^Tg/Tg^ Rag1^−/−^ mouse was crossed with the TDLacZ^Δ/Δ^ Rag1^−/−^ mouse heterozygote for TCR Tg and TDLacZ, only CD4^−^ CD8^−^ DN cells were detected in the smaller thymus (Figure 7). The total number of thymocytes recovered was approximately 17.5% of what was recovered from a TCR Tg mouse. Most of the cells were arrested at the CD25^hi^ CD44^−^ (DN3) stage, similar to what was observed in a Rag1^−/−^ mouse (Figure 7B). However, massive cell death in the DN as well as CD4 and/or CD8 cells was found only in the BgII^Tg+^ TDLacZ^Δ/+^ mouse (Figure 7C). By contrast, in the BgII Tg alone, the fraction of CD44^−^ CD25^−^ (DN4) cells had the highest DN subpopulation (Figure 7B). Thus, in the presence of LacZ, the TCR Tg thymocytes appeared to be substantially deleted at the post-DN3 stage as soon as they expressed their TCR at the cell surface (see Figures S2 and S3 for the expression of TCR gene and protein on the surface).

The pattern of thymic stromal cells (gated on CD45^−^ cells) observed in the BgII^Tg/+^ TDLacZ^Δ/+^ Rag1^−/−^ mice was also similar to that of a Rag1^−/−^ mouse (Figure 7D). UEA1^+^ mTECs, which are prominent in the adult wild-type thymus, were barely detected, and thus the proportion of CDR1^+^ cTECs was greatly elevated. Therefore, BgII^Tg/+^ TDLacZ^Δ/+^ Rag1^−/−^ mice do not harbor fully developed mTECs, yet they remain able to efficiently delete developing thymocytes.

Cross-Presentation by DC or mTEC Is Not Involved in CDR1^+^cTEC-Mediated Deletion

**Figure 8:**
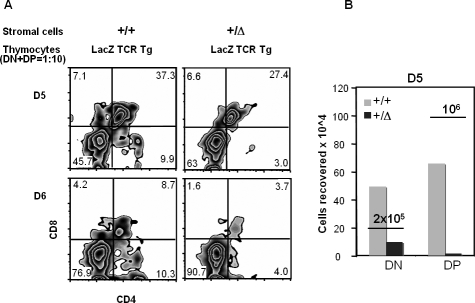
DC and mTEC Are Not Necessary for the Deletion of Transgenic Thymocytes Sorted anti-LacZ TCR thymocytes were reaggregated with UEA-1–depleted CD45^−^ stromal cells from dGuo-treated fetal thymuses. (A) Flow cytometric profiles of reaggregate cultures initiated with 3.8 × 10^5^ DN + DP (1:10 ratio) and 5 × 10^5^ purified thymic stroma at day 5 (top) or day 6 (bottom). The sources of stromal cells and thymocytes are indicated at the top of the graphs. CD4 and CD8 profiles are shown along with the percentage of cells in each of the compartments. Typically, cell recoveries of the DN and DP thymocyte mixtures cultured with TDLacZ heterozygote (+/Δ) stroma were 20%–30% of thymocyte mixtures cultured with wild-type (+/+) stroma. (B) A representative cell recovery when DN and DP cells were cultured separately with purified stroma from the wild type (+/+) versus the heterozygote (+/Δ ) mouse. Underlined numbers indicate the initial numbers of cells that were input.

In order to exclude the possibility of cross-presentation by mTEC and DC in the induction of tolerance, we employed a clean reaggregate thymic organ culture system (RTOC) [49,50] using UEA-1– and CD45-depleted thymic stroma reconstituted with purified anti-LacZ TCR transgenic thymocytes. The stromal cells were prepared from a E14.5 fetal thymic organ culture, in the presence of 2-dGuo, which depletes DC, and the population was further depleted of CD45^+^ and UEA-1^+^ cells by magnetic bead separation. Using such cells isolated from wild-type or TDLacZ^+/Δ^ thymus samples, anti-LacZ TCR-bearing DN and DP cells (1:10 ratio, similar to that of a normal thymus) from adult BgII^Tg/Tg^ animals were reaggregated with them and cultured for 5–6 d. As shown in Figure 7, the recovery of the thymocytes from the RTOC with TDLacZ cTECs was between 5%–20% of that achieved with the wild-type stroma. In addition, these cultures did not contain a significant number of CD4 single-positive (SP) cells (Figure 8A). When the DN and DP transgenic thymocytes were separately reaggregated with TDLacZ cTEC (Figure 8B), both subsets showed reduced cell numbers after culture, indicating that LacZ expression could also deplete the LacZ-responding DP cells in the RTOC. Although these results argue against the idea of impaired differentiation from the DN to the DP stage, they suggest that developing thymocytes are deleted once they react with antigens presented in thymic cortical epithelium.

## Discussion

In this report, we have examined the induction of tolerance to a TSCOT^+^ TEC subset-specific antigen. Our results demonstrate that a small amount of antigen (a few thousand/cell), present in a small number of TEC subset (4,000 cells/thymus), functions as a highly efficient tolerogen in midst of diverse repertoire. In addition, cortical marker CDR1^+^ TEC delete TCR^+^ Tg thymocytes without cross-presentation by either DC or mTEC. This type of tolerance was established, at least, partially in an AIRE-independent manner.

### Tolerance by the TSCOT^+^CDR1^+^MHCII^hi^ Compartment in the Thymic Microenvironment

From the histological analyses, the thymic microenvironment is already known to be complex in nature, and to change during development [41,51–53]. In addition, histological analyses alone do not constitute a suitable method for delineating expression profiles for different compartments, because of the poor cortical staining [43]. Using a combination of flow cytometry with compartment-specific markers [43] and LacZ reporter staining, cortical expression of the TSCOT locus was confirmed at the newborn stage (Figure 2A and [37,41,51–53]). In the mature thymus, β-gal activity was also principally found in the cortex (Figure 2B), and TSCOT surface expression was exclusively detected in CDR1^+^ TEC populations, not in conventional mTEC or nonepithelial cells (Figure 2C). However, there are cases in which LacZ activity staining extends to the corticomedullary junction of mature TDLacZ thymus and TSCOT marker also stains uncharacterized minor population of cells (unpublished data). A part of these minor populations could be developing transitional TEC, but this possibility requires further detailed study with an improved technology that can handle an extremely small number of cells. Nonetheless, it is clear that TSCOT^+^ cells are not part of conventional medullary cells. TSCOT/LacZ was never detected in the conventional CDR1^−^UEA-1^+^ mTEC population (Figure 2C). Thus, we are able to dismiss the possibility that LacZ is ectopically expressed in the mTEC of the medulla. Furthermore, TSCOT expression was widely located in the Rag1^−/−^ thymus [38], which lacks mature mTEC (Figure 7C and [54]). In case of BgII mouse on a Rag1^−/−^ background, tolerance at the DN stage was very clear when there was antigen only in the cortical epithelium (Figure 7).

The notion of the exclusion of cortical epithelium in the induction of tolerance was derived from the transgenic expression of MHC molecules exclusively in the cortex of the thymus, using the K14 promoter [25,26]. However, the idea of an exclusive tolerance niche has been challenged: incomplete, but significant, tolerance was observed when other antigens were targeted to cTEC using the same promoter [27,28]. In addition, it has been clearly demonstrated that the K14-MHCII thymus is in fact autotoleragenic when self-antigens are presented by its own cortical epithelial cells [22]. Our current findings using a specific TSCOT promoter corroborate the notion that cTEC participate in the establishment of CD4 central tolerance in a highly efficient manner. There is no evidence for autoreactivity to specific antigens presented by the TSCOT^+^ cells. Instead, we found clear tolerance to LacZ antigen. Therefore, the TSCOT^+^ TEC niche of the cTEC subpopulation is not excluded from the tolerance induction so that it may avoid autoreactivity against its own cell.

### The Mechanism of Tolerance Induction by the TSCOT^+^ TEC Subset

Taking advantage of sensitive enzymatic activity, our estimate for LacZ under the control of the TSCOT promoter is about 6,000 (the average of two measurements) molecules per TSCOT^+^ TEC in homozygote thymus (Figure 3). This number is surprisingly similar to that of the estimation of mTEC derived from the indirect estimation [55]. However, one half of this amount in heterozygotes was sufficient to induce complete CD4 tolerance in the absence of mTEC (Figures 7 and 8) or DC cross-presentation (Figure 8). Previous accurate estimates [56] have suggested that recognition of only three to four peptide/MHC complexes by an immature thymocyte was sufficient to generate a negative selection event in transgenic mouse. Therefore, it remains a challenging question as to how such a high efficiency is achieved. The number of cTEC in the adult thymus is far less than that of mTEC [43]. The total number of TSCOT^+^ TEC, estimated from a large pool of adult thymuses, was only on the order of several thousand per thymus. In order to screen all of the developing thymocytes for potential autoreactivity, the frequency of cell encounters between cTEC presenting the specific antigen and thymocytes would have to be optimized, even considering the average 3-d period in which DP thymocytes remain in the cortex [57,58]. This could be accomplished in thymic nurse cells in which multiple thymocytes are found in association with one epithelial cell.

Several earlier papers had come to the conclusion that the thymic epithelium induced tolerance by the induction of anergy, rather than deletion [59,60]. In contrast, in our anti-LacZ TCR Tg × TDLacZ model, it is evident that deletion is the dominant mechanism (Figure 7). Deletion has also been observed in a number of other TCR transgenic systems [28,61]. Whether this is a normal physiological process or death subsequent to developmental arrest following the premature expression of a transgenic receptor at the DN stage was questioned [62]. However, DP thymocytes included in the RTOC were also deleted, arguing that cTEC-induced tolerance involves the specific deletion rather than the arrest at premature developmental stage (Figure 8B).

In the TDLacZ thymus, cortical epithelium expressing a specific antigen was able to tolerize quite thoroughly (Figures 7 and 8). This was somewhat surprising if thymic epithelial cells are poor presenters of antigen as concluded earlier on the poor expression of costimulatory molecules in cTEC [63]. However, we clearly show that TSCOT^+^ cTEC express high levels of MHCII, CD54, CD40, and CD80. Surface CD40 and CD80 proteins in particular are expressed at surprising levels that are even higher than those of mTEC (Figure 6). According to the RT-PCR result from the purified cells, CD40, CD80, and CD86 message levels are slightly higher in mTEC than cTEC ([37] and unpublished data). Among cTEC, TSCOT^+^ cells are the ones expressing more costimulatory molecules (Figures 6 and S1). Therefore, molecules on the TSCOT^+^cTEC can provide the environment for the highly efficient deletional tolerance of TCR bearing early thymocytes Figure S2) through a unique TSCOT^+^ cTEC antigen presentation process. As seen in figure 7C, massive apoptosis events in DN TCR transgenic cells in the presence of the LacZ antigen-bearing cTEC are also consistent with the deletional tolerance.

In order to determine the molecular mechanism underlying the induction of tolerance, we determined whether or not AIRE was involved. It has been fairly well established that AIRE is involved in mTEC-mediated tolerance induction by facilitating the expression of peripheral antigens in normal and genetically modified animals [12,64,65]. As a result of the introduction of one copy of the LacZ gene into AIRE-deficient animals, the LacZ-specific CD4 proliferative response was significantly reduced (Figure 5). Since AIRE expression was, mostly, assumed to be absent in the normal CDR1^+^ cTEC (except Rag1^−/−^), it is not surprising that AIRE was not directly involved in TSCOT^+^ TEC-induced tolerance. Instead, it may suggest that the AIRE-independent tolerance pathway exists in the TSCOT^+^ TEC. However, there was still the question of whether tolerance was induced by AIRE-expressing DC or mTEC, via a cross-presentation. The results obtained with RTOC using thymocytes from LacZ-specific TCR transgenic mice and purified CD45^−^UEA-1^−^CDR1^+^ cells from 2-dGuo–treated FTOC (Figure 7) show that neither DC nor mTEC are necessary for tolerance induction in vitro. The direct involvement of TSCOT^+^ TEC in deletional tolerance constitutes strong evidence for the capacity of direct antigen presentation [29,32–36]. More detailed studies will be required to identify the specific molecules that are involved in this type of antigen presentation.

### Are Affinity/Avidity Models Sufficient to Explain Central Tolerance by Cortical Epithelium?

It is generally accepted that negative selection requires specific conditions of either high-avidity interaction or prolonged signaling [20,66,67]. The quantitative aspects discussed above seem insufficient to explain negative selection by a simple affinity/avidity model for cTEC. The surface and cytoplasmic levels of MHCII in cTEC are not appreciably lower than in mTEC and MHCII molecules exist on cTEC as aggregates on the surface [43]. Thus, if a self-peptide was presented at sufficiently high concentrations to display multiple complexes in the same aggregate at any one time, these MHCII aggregates could potentially generate high-avidity signaling leading to thymocyte death. If so, then cortical epithelium could function directly in both negative and positive selection. In line with this notion, it has been shown that a single cTEC line can mediate both positive and negative selection [68]. If the amount of any antigen produced by a cTEC is low, then under normal conditions with a random loading mechanism for a diverse set of endogenous peptides [69], a single MHCII aggregate on the cTEC surface would likely contain only one peptide/MHC complex. This would hinder an avidity-based mechanism from operating as there would be no multimeric presentation. Although such monomeric presentation might be adequate for positive selection, it seems that it would be inadequate for negative selection. This raises the possibility that other mechanisms might exist for increasing the antigen density on cTEC. Such a mechanism might involve intercellular antigen transfer [70], in addition to sampling of other self-antigen pools [8,71]. However, the expression of costimulatory molecules on TSCOT^+^ cTECs is consistent with the idea that the presence of costimulation/second signals may distinguish negative from positive selection.

## Materials and Methods

### Mice.

All mice were handled according to American Association for Accreditation of Laboratory Animal Care Regulations.

In order to generate mice carrying an inserted *LacZ* allele at the TSCOT locus, a 4.2-kb targeting vector was constructed by cloning IRES-LacZ with a neo-selectable marker from p1049 [42] between two BamHI sites in the first exon of the *TSCOT* gene. A Herpes Simplex Virus thymidine kinase (HSV-TK) expression cassette was positioned at the 5′ end of the construct in order to facilitate negative selection for homologous recombination. The targeted allele harbors an IRES-LacZ and PGK-neomycin expression cassette within the first exon, resulting in a small deletion (284 bp) between the two BamHI sites within exon 1. The mouse 129 embryonic stem cell line (R1) was electroporated with the construct, and the neomycin-resistant clones were screened in the laboratory of Dr. Hua Gu (National Institute of Allergy and Infectious Diseases [NIAID]/ National Institutes of Health [NIH]). Chimeras were generated by blastocyst injection, and one founder mouse was backcrossed to C57BL/6Tac.

To study antigen-specific CD4^+^ T cell responses to β-gal, a transgenic mouse strain on a C57BL/6 background was developed and named BgII. RNA was isolated from an I-Ab–restricted, β-gal–specific CD4+ T cell clone. Total mRNA was isolated using a Qiagen RNeasy kit, and the α and β TCR were amplified by 5′-Rapid Amplification of cDNA Ends (5′-RACE, Life Technologies) using constant region anti-sense primers a1 (5′-GGCTACTT TCAGCAGGAGGA-3′) and b1 (5′-AGGCCTCTGCACTGATGTTC-3′), respectively. The 5′-RACE products were amplified with nested TCR α and β constant region primers a2 (5′-GGGAGTCAAAGTCGGTGAAC-3′) and b2 (5′-CCACGTGGTCAGGGAAGAAG-3′), and cloned into pCR4TOPO TA sequencing vectors (Invitrogen). Genomic cloning PCR primers were designed based upon the method previously described [72]. The genomic variable domains were TA cloned into pCR4TOPO (Invitrogen), validated by sequencing, subcloned into TCR cassette vectors kindly provided by Dr. Diane Mathis (Harvard), and coinjected into fertilized C57BL/6 embryos (SAIC) yielding TCR transgenic founder which were then bred.

PCR genotyping.

Tail or ear samples were employed for genotyping, using the red Extract-N-Amp Tissue PCR kit (Sigma) and primers: for the TSCOT locus, Neo primer: ACCGCTATCAGGACATAGCGTTGG, 1C12 F1: TTACTCAAAGTGATGCTGGACTGG, 1C12 B2: CCGAGGGTTCCTTGGTACATTC; for the RAG1 locus, Neo primer: ACCGCTATCAGGACATAGCGTTGG, Rag-1 F: TCGTTTCAAGAGTGACGGGCAC, Rag-1 B: AATCCTGGCAATGAGGTCTGG; and for the AIRE locus, forward primer: GTCATGTTGACGGATCCAGGGTAGAAAGT, reverse primer: AGACTAGGTGTTCCCTCCCAACCTCAG. For the anti-LacZ TCR transgenic allele, BG2 Alpha F1: ACAACCCGGGATTGGACAG, BG2 Alpha R1: GTATAGCGGCCGCCTCCTAGTGCAATGGT, BG2 Beta F1: TATCTCGAGTCCTGCCGTGACCCTACTATG; BG2 Beta R1: CAGCCGCGGAACCCAACACAAAAACTATAC.

### Flow cytometry.

Antibodies used for flow cytometric analysis were as follows: for stromal cells, FITC-conjugated anti-mouse I-A^b^ (A^b^) AF6–120.1 (BD Pharmingen), CDR1-PE (prepared by L. Lantz, NIAID flow cytometric facility), CD45 PE-Texas Red conjugate (Caltag), biotinylated Ulex europaeus agglutinin-1 (Vector Laboratories), streptavidin-APC (BD Pharmingen), CLVE1 anti-TSCOT mAb (prepared by Dr. L. Lanz, NIAID), and FITC-conjugated goat anti-Rat IgM (Jackson Laboratories). For thymocytes, FITC-conjugated anti-mouse CD4 (L3T4) (GK1.5) (BD Pharmingen), PE-conjugated anti-mouse CD44 (BD Pharmingen), mouse CD8α PE-Texas Red conjugate (Caltag), biotin-conjugated anti-mouse CD25 (BD Pharmingen), streptavidin-APC (BD Pharmingen), annexinV-FITC (Clontech), PE-conjugated anti-mouse CD69 (BD Pharmingen), CD4 PE-Texas Red conjugate (Caltag), biotin anti-mouse αβTCR (H57–597) (BD Pharmingen), FITC-conjugated anti-mouse CD44 (BD Pharmingen), PE-conjugated anti-mouse CD25 (BD Pharmingen), APC-conjugated anti-mouse CD4 (L3T4) (GK1.5) (BD Pharmingen), and APC-conjugated anti-mouse CD8α (BD Pharmingen). For LacZ staining, after treating with 30 mM chloroquine diphosphate to block endogenous β-gal activity for 30 min at 37 °C, 33 μM ImaGene Red C12RG substrate (Molecular Probes, ImaGene Red C12RG lacZ Gene Expression Kit I-2906) was used in FACS buffer for 20 min at 4 °C.

### β-galactosidase activity and antibody staining.

Either whole embryos or isolated thymuses were washed in PBS and fixed in 1% paraformaldehyde, 0.2% glutaraldehyde, 0.02% NP-40, 1 mM MgCl_2_ in PBS for 1 or 2 h on ice. Staining was conducted using X-gal solution with 100 mM d-galactose in 2 mM MgCl_2_, 5 mM potassium ferricyanide, 5 mM potassium ferrocyanide overnight at 37 °C [42]. For the sections, the thymuses were embedded in Tissue Freezing Medium (Triangle Biomedical Sciences). The 4-μm sections were fixed for 2 min in 1% formaldehyde, 0.2% glutaraldehyde, 0.02% NP-40 1 mM NaCl, then incubated with X-gal solution (1 part X-gal 40 mg/ml in dimethyl formamide, in 40 parts 2 mM MgCl_2_, 5 mM potassium ferricyanide, 5 mM potassium ferrocyanide in PBS) at 37 °C for 48 h. For antibody staining, the paraffin sections were stained with DAKO CSA reagent. For the LacZ Lysis assays, thymic cells from 30 TDLacZ mice or control C57BL/6 mice were partially purified via MACS CD45-bead sorting (Miltenyi Biotech). The cell pellets were lysed using Reporter Lysis Buffer from the β-Galactosidase Enzyme Assay System with Reporter Lysis Buffer (Promega). After lysis and centrifugation, the Assay Buffer was added to each supernatant as well as to enzyme aliquots for a standard curve, and then incubated for 30 min at 37 °C. Absorbance was then measured at a wavelength of 405 nm.

### Cytospin and confocal microscopy.

Isolated TEC (about 10^5^) were washed in cold FACS buffer (PBS + 1% BSA), subsequently stained on ice with 2.4G2, APC-conjugated anti-CDR1, biotinylated anti-CD80 (B7–1, Armenian hamster IgG2κ) followed by streptabidin-Alexa568 (Molecular Probes). For the detection of LacZ-expressing cells, ImaGene Green C_12_FDG lacZ Gene Expression kit (Molecular Probes) was used. Stained samples were placed on the slides by cytospin at 1,200 rpm for 2 min. Images were collected on LSM 510 META (Zeiss), and analyzed with LSM Image Examiner (Zeiss) and Photoshop.

### CD4^+^ T cell in vitro proliferation assay.

Three mice per group were immunized with the affinity-purified, LPS-removed recombinant proteins in Complete Freund's Adjuvant in one footpad and the base of the tail. Ten days after immunization, the mice were sacrificed and the inguinal, mesenteric, and para-aortic lymph nodes were collected and crushed in Iscov's Modified Dulbecco's Medium. The CD4^+^ cells were collected from MACS columns using anti-CD4 antibody (GK1.5) (purity of the cells were usually over 95%) and incubated at 37 °C with irradiated whole spleen cells and 0, 1, 10, or 100 μg of LacZ protein (in triplicate). The cells were pulsed with ^3^H-thymidine for the final 24 h of a 72-h incubation. The cells were harvested with a Brandel 96-well harvester, and thymidine incorporation into DNA was measured with a Wallac Trilux 1450 β-scintillation counter.

### Detection of antibodies to LacZ by ELISA.

The mice were immunized intraperitoneally with either LacZ or purified recombinant GST-TSCOT-Loop protein in CFA, three times every other week. The mice were bled 3 d after the last injection, and the sera were incubated on His-LacZ protein or His-Loop–coated (5 μg/well) ELISA plates. The bound anti-LacZ Ab was detected with anti-mouse immunoglobulin isotype-specific antibodies conjugated with HRP and assayed with ABTS solution (Southern Biotechnology Associates) by following the manufacturer's description. Optical density (OD) was measured at 405 nm.

### Reaggregate thymic organ culture (Anderson and Jenkinson Protocol).

The thymic stromal cells were prepared by treating E14.5 fetal thymus samples with 2-dGuo for a week. UEA-1^−^ and CD45^−^ cells were then purified using biotinylated reagents and streptavidin-MACS beads. Thymocytes from anti-LacZ TCR transgenic mice were sorted for DP and DN cells and reaggregated with the stromal cells using protocols developed by Anderson and Jenkinson at the University of Birmingham, United Kingdom [49]. Recovered cells were counted and then analyzed by flow cytometry after 4–6 d of culture.

## Supporting Information

Figure S1 Quantitative CD80 Analysis of mTEC, TSCOT^+^ cTEC, and TSCOT^−^ cTEC The analysis was performed using confocal microscopic data. Levels of CD80 were measured using the sum of intensities in the cell area over the background (TINA2.09f, Pusan National University). Percent of maximum (max) was calculated with an average of three CDR1^+^LacZ^+^ cells. Bars and dots represent the average and each values, respectively. Asterisk indicates area from the adjacent cell was excluded for the measurement.
(2.09 MB TIF)

Figure S2 Genotyping of TCR Transgenic Mouse Genotyping was done as described in Materials and Methods. Copy of the relevant page for the mouse information and the genotyping results are shown.
(1.60 MB TIF)

Figure S3 Expression of Transgenic TCR during Thymocyte Development TCR levels were detected with anti-TCRb chain antibody (H57) in combination with other antibodies in flow cytometry. CD4 and CD8 profiles, and DN, DP, and CD4 gates are shown in the top left panel; TCR levels of respective gates are shown on the upper right. DN gated CD44 and CD25 profiles, and DN1, DN2+3, and DN4 gates are shown on the lower left. TCR levels of respective DN gates are shown on the lower right. Solid lines in the histogram show the TCR levels of each gate in the CD4 TCR transgenic mouse; dotted lines show those of the same gated in the wild-type B6 mice.
(3.35 MB TIF)

## Abbreviations

APC: antigen-presenting cell
cTEC: cortical thymic epithelial cell
DC: dendritic cell
DN: double negative
DP: double positive
E: embryonic day
mAb: monoclonal antibody
MHC: major histocompatibility complex
mTEC: medullary thymic epithelial cell
RTOC: reaggregate thymic organ culture
TDLacZ: thymic stromal cotransporter delta LacZ
TCR: T cell receptor
TEC: thymic epithelial cell
TSCOT: thymic stromal cotransporter
β-gal: β-galactosidase
